# Cross-sectional associations between genetic polymorphisms in metabolic enzymes and longer leukocyte telomere length induced by omethoate

**DOI:** 10.18632/oncotarget.20971

**Published:** 2017-09-18

**Authors:** Xiaoran Duan, Yongli Yang, Sihua Wang, Xiaolei Feng, Tuanwei Wang, Pengpeng Wang, Suxiang Liu, Lei Li, Guoyu Li, Wu Yao, Liuxin Cui, Wei Wang

**Affiliations:** ^1^ Department of Occupational and Environmental Health, College of Public Health, Zhengzhou University, Zhengzhou, China; ^2^ Department of Epidemiology and Biostatistics, College of Public Health, Zhengzhou University, Zhengzhou, China; ^3^ Department of Occupational Health, Henan Institute for Occupational Medicine, Zhengzhou, China; ^4^ Clinical Department, Zhengzhou Institute of Occupational Health, Zhengzhou, China

**Keywords:** omethoate, telomere, polymorphism, metabolic enzymes

## Abstract

**Purpose:**

This study aimed to explore the effects of genetic polymorphisms in metabolic enzymes on relative telomere length changes and explore the mechanism of canceration induced by omethoate.

**Materials and Methods:**

180 long-term omethoate-exposed workers and 115 healthy controls were recruited. Real-time PCR method was applied to determine the relative telomere length in peripheral blood leukocytes DNA, and Six polymorphic loci of *GSTT1*(+/−), *GSTM1*(+/−), *GSTP1* rs1695, *CYP2E1* rs6413432, *CYP2E1* rs3813867 and *PON2* rs12026 were detected by polymerase chain reaction and restriction fragment length polymorphism method; Multiple linear regression was conducted to explore the effects of omethoate exposure and genetic polymorphisms on the telomere length.

**Results:**

The relative telomere lengths in the control group (0.94 [0.76, 1.32]) were significantly shorter than that in the exposure group (1.50 [1.11, 2.57]) (*Z* = 7.910, *P* < 0.001). Univariate analysis showed that the relative telomere lengths of the *GSTM1*-deletion individuals were significantly longer than that of the non - deletion genotype in the control group (*Z* = 2.911, *P* = 0.004), and the relative telomere lengths of *GSTP1* rs1695 polymorphism locus (GG+AG) genotype individuals were longer than that of AA genotype in the exposure group. The difference was statistically significant (*Z* = 2.262, *P* = 0.024). Multivariate analysis found that pesticide-exposure (*b* = 0.524, *P* < 0.001) and GSTM1 polymorphism (*b* = −0.136, *P* = 0.029) had an impact on telomere length.

**Conclusions:**

The relative telomere lengths of omethoate-exposure workers were longer than that in the control population. Also *GSTM1* genetic polymorphism may influence the changes of the telomere length induced by omethoate.

## INTRODUCTION

Omethoate is an organophosphorous insecticide and acaricide that is highly toxic, highly effective and broad-spectrum features. In the process of its use and production, it can cause certain harm to the health of persons who come into contact with it, such as chronic toxicity caused by long-term, low-dose exposure which is easy to be ignored [1, 2].

In recent years, acute organophosphorous poisoning incidents have been gradually reduced, and people are more concerned about the chronic toxic effect caused by long-term, low-dose exposure to OPs. More and more studies have concluded that there was a close relationship between long-term, low-dose exposure Ops and many human diseases, such as tumorigenesis, adverse reproductive outcomes, and neurological and neurobehavioral function abnormalities [3–5]. Studies on organophosphorous pesticides involving genetic damage mainly included comet assay, micronucles (MN), and sister chromatid exchange (SCE) [6–11]. However, it was rarely reported that OPs induced telomere damage. Telomere is specialized DNA-protein functional complex which is located at the end of the chromosome and is more susceptible to be attacked by exogenous compounds, telomere excessive consumption or structural disruption can result in cell carcinogenesis [12]. Therefore, telomere length can be used as an effect marker for analysis and research.

Organophosphorous pesticides experience a complex metabolic process after entering into the body. A wide variety of enzymes are involved in the organophosphorus metabolism, including the active enzyme in the phase I reaction and the binding enzyme in the phase II reaction. The activity of enzymes and the metabolic level are different for each person; therefore, not all individuals will produce genetic damage under the same contact conditions, suggesting that genetic susceptibility is different for individuals who are exposed to an organophosphorus pesticide. At present, much attention has been given to the five functional genes *GSTM1, GSTT1, GSTP1, CYP2E1, and PON2* polymorphisms. Enzymes encoded by these genes are involved in metabolism and detoxification of exogenous substances. Studies have shown that the *GSTM1* null genotype could increase the tail moment of the comet assay in workers occupationally exposed to OPs [13]. A previous study performed in pesticide-exposed fruit growers also showed that the mutant genotype of *GSTP1* rs1695 polymorphism locus was associated with an increased risk of DNA damage measured by the comet assay [14]. Wong et al [15] researched the relationship between *XRCC1, CYP2E1* and *ALDH2* genetic polymorphisms and the sister chromatid exchange (SCE) frequency and found that *CYP2E1* rs3813867 mutant genotype CC could increase SCE frequency. Paraoxonase2 (*PON2*) is a member of the paraoxonase gene family which can protect cells from oxidative stress and thus protect chromosomes from damage. Variants of rs12026 within the *PON2* gene were associated with cardiovascular disease, cerebrovascular disease, diabetes and other diseases [16, 17]. However, the relationship between these genetic polymorphisms and telomere length in workers exposed to omethoate has not been reported.

Therefore, we studied telomere length changes in workers exposed to omethoate and the effects of the above five metabolism genetic polymorphisms.

## RESULTS

### Demographic characteristics of the study population

Table 1 showed the compared results of gender, age, smoking and drinking history between the two groups. Gender, age, smoking and drinking history had significant differences between the two groups. We regarded 40 years old as the boundary and divided the ages into the low and high age group according to the segments of Chinese age.

**Table 1:** General characteristics of exposure and control groups (%)

Variable	Exposure	Control	Total	χ2	*P*
Gender					
Male	137 (76.1)	54 (47.0)	191 (64.7)	26.130	< 0.001
Female	43 (23.9)	61 (53.0)	104 (35.3)		
Age(years)					
≤ 40	53 (29.4)	67 (58.3)	120 (40.7)	24.146	< 0.001
> 40	127 (70.6)	48 (41.7)	175 (59.3)		
Smoking					
Yes	63 (35.0)	12 (10.4)	75 (25.4)	22.333	< 0.001
No	117 (65.0)	103 (89.6)	220 (74.6)		
Drinking					
Yes	16 (8.9)	30 (26.1)	46 (15.6)	15.769	< 0.001
No	164 (91.1)	85 (73.9)	249 (84.4)		

### The detecting results of external exposure

According to the detecting reports from 2011 to 2013, the 8 h time-weighted average and short-term exposure concentrations were lower than occupational exposure limits prescribed by the state in each type of work.

### The determination results of relative telomere length

Statistical analysis found that the relative telomere lengths in the normal controls (0.94 [0.76, 1.32]) were significantly shorter than that of the exposed group (1.50 [1.11, 2.57]) (Z = 7.910, *P* < 0.001).

### Effects of gender, age, smoking, drinking and working duration on telomere length

To analyze the correlation between age and telomere length using the Spearman rank correlation, we found that there was no correlation in the exposure or control group, respectively (*r_s_* = 0.049, *P* = 0.517; *r_s_* = 0 .116, *P* = 0.218). Table 2 showed that gender, age, smoking, drinking and working duration had no effect on the relative telomere length in the exposure or control group(*P* > 0.05). However, the relative telomere lengths were different between the two groups in the same stratification, and the difference were statistically significant (*P* < 0.05).

**Table 2:** The effects of sex, age, smoking, drinking and working duration on telomere length

Variables	Exposure		Control		Z*	P*
	*n*	*M* (*P*_25_, *P*_75_)	*n*	*M* (*P*_25_, *P*_75_)		
Gender						
Male	137	1.50 (1.10,2.57)	54	0.93 (0.77,1.16)	6.871	< 0.001
Female	43	1.79 (1.11,2.90)	61	1.08 (0.74,1.48)	4.360	< 0.001
Z^#^		0.787		1.569		
*P*^#^		0.431		0.117		
Age						
≤ 40	53	1.50 (1.26,2.44)	67	0.95 (0.77,1.32)	5.356	< 0.001
> 40	127	1.54 (1.06,2.62)	48	0.94 (0.73,1.34)	5.568	< 0.001
Z^#^		-0.328		−0.556		
*P*^#^		0.743		0.578		
Smoking						
Yes	63	1.50 (1.08,2.48)	12	1.20 (0.94,1.33)	2.298	0.022
No	117	1.54 (1.13,2.66)	103	0.94 (0.74,1.32)	7.254	< 0.001
*Z#*		-0.321		-1.409		
*P*^#^		0.748		0.159		
Drinking						
Yes	16	1.50 (1.20,2.22)	30	0.92 (0.76,1.28)	3.759	< 0.001
No	164	1.50 (1.10,2.61)	85	0.95 (0.76,1.35)	6.753	< 0.001
Z^#^		−0.065		−0.293		
*P*^#^		0.948		0.770		
Working duration						
< 15	26	1.61 (1.23,2.70)				
15∼30	117	1.54 (1.12,2.72)				
> 30	37	1.35 (0.99,2.20)				
χ*2*^#^		2.251				
*P*^#^		0.324				

Note: P* indicates the comparison of telomere length between exposure group and control group after stratifying, *P*^#^ represents the comparison among the layers after stratifying.

### Effects of genetic polymorphisms on telomere lengths

The genotype distribution in these loci conformed to the Hardy-Weinberg balance (*P* > 0.05), suggesting the control samples had representativeness. To regard wild homozygous genotype as a reference, we analyzed the differences of relative telomere length between heterozygous or mutant homozygous genotype and wild homozygous genotype. Among them, the wild homozygous genotype of *GSTP1*rs1695 locus and mutation homozygous genotype of *PON2* rs12026 locus were 2 cases and 3 cases respectively in the exposure group; therefore, they would be a merger with the heterozygous genotype directly. The relationships between genetic polymorphisms and telomere lengths are shown in Table 3. The results show that the relative telomere length of the *GSTM1* polymorphism deletion genotype was significantly longer than that of the non-deletion genotype in the control group (*Z* =2.911, *P* = 0.004), and the relative telomere length of GG+AG genotypes for *GSTP1* rs1695 polymorphism locus was statistically significant longer than that of the AA genotype in the exposure group (Z = 2.262, *P* = 0.024), and the genotypes of other loci had no statistically significant differences (*P* > 0.05).

**Table 3:** The relationships between genetic polymorphism and telomere lengths

SNPs	Exposure				Control			
	*n*	*M* (*P*_25_, *P*_75_)	*Z*	P**	*n*	*M* (*P*_25_, *P*_75_)	*Z*	P**
*GSTT1*								
+	99	1.50 (1.06,2.69)	0.653	0.514	67	0.94 (0.74,1.27)	1.100	0.271
−	81	1.50 (1.19,2.43)			48	1.04 (0.77,1.45)		
*GSTM1*								
+	85	1.42 (1.07,2.14)	1.266	0.205	51	0.91 (0.71,1.08)	2.911	0.004
−	95	1.60 (1.16,2.91)			64	1.12 (0.77,1.47)		
*GSTP1 rs1695*								
GG+AG	54	1.73 (1.30,3.16)	2.262	0.024	44	0.94 (0.78,1.34)	0.253	0.800
AA	126	1.46 (1.06,2.24)			71	0.94 (0.75,1.32)		
*CYP2E1*rs6413432								
TT	97	1.50 (1.15,2.37)	Ref*		68	0.94 (0.74,1.33)	Ref*	
AT	72	1.50 (1.02,2.97)	0.286	0.775	42	1.04 (0.79,1.29)	0.381	0.704
AA	11	1.64 (1.05,3.17)	0.276	0.782	5	0.71 (0.65,1.21)	1.157	0.247
^χ2^ (P*)		0.200 (0.905)				1.668 (0.434)		
*CYP2E1*rs3813867								
GG	106	1.50 (1.13,2.46)	Ref*		60	0.95 (0.73,1.35)	Ref*	
CG	65	1.50 (1.03,2.70)	0.395	0.693	50	1.04 (0.79,1.30)	0.381	0.703
CC	9	1.64 (1.11,4.16)	0.698	0.485	5	0.71 (0.65,1.00)	1.613	0.107
^χ2^ (P*)		0.760 (0.684)				3.083 (0.214)		
*PON2* rs12026								
CC	125	1.48 (1.08,2.53)	1.099	0.272	62	0.94 (0.74,1.34)	0.443	0.658
CG+GG	55	1.66 (1.20,2.74)			53	0.95 (0.76,1.32)		

P*: To compare telomere lengths among genotypes using a rank test of k independent samples; P**: The comparing results between the two groups, the Bonferroni method was adopted; * Ref: The reference group of comparing different genotypes.

### The effects of risk factors on relative telomere length in omethoate-exposed workers

The influencing factors were screened using multiple linear regression. The data of telomere length was logarithmically transformed to meet the conditions of multiple linear regression analysis because of its abnormal distribution. Ln (natural logarithm) was established (Telomere length) as the dependent variable, and the independent variables were dummy variables, and screen the independent variables using a stepwise method. The variables kept in the models included group (*b* = 0.524, *P* < 0.001), *GSTM1* gene polymorphism (*b* = −0,136, *P* = 0.029). No found that gender, alcohol, smoking, age, working duration and *GSTT1*, *GSTP1* rs1695, *CYP2E1* rs6413432, *CYP2E1* rs3813867, *PON2* rs12026 genetic polymorphism were included in the model. Table 4 showed the results.

**Table 4:** Independent variables of entering the regression model

Variables	Unstandardized Coefficient	Standard Error	Standardized Coefficient	*t*	*P*
Constant term	0.061	0. 057		1.083	0.280
*Exposure category*	0.524	0.063	0.433	8.266	< 0.001
*GSTM1*	−0.136	0.062	−0.115	−2.199	0.029

## DISCUSSION

Telomeres are specialized DNA-protein structures at the ends of the linear chromosomes. Their role is to protect the integrity and stability of the chromosome [12, 18]. At present, the results in a large number of studies showed that telomeres and telomerase abnormalities were related to tumor formation; however, Professor Carol Greider has proposed that telomerase was not able to explain why telomeres have a certain length and how to extend the length of telomeres, and longer telomeres were often directly related to cancer [19].

At present, the research results were different in the changes of telomere length caused by poisons; many studies suggested that different toxicants could cause telomere shortening [20, 21]. However, some studies found that toxicants could extend telomere length [22, 23]. Elongated telomere length has been observed as an early response after low-dose chemical carcinogens *in vitro* and animal experiments, suggesting low-dose carcinogenic chemical exposure may function as a tumor promoter at the early stage of human carcinogenesis [22]. This cross-sectional study also found that relative telomere lengths in the exposure population were significantly longer than that of the control (*Z* = −7.910, *P* < 0.001); And we found that the relative telomere length decreased with the extension of working duration in the exposure group. The assessment of the occupational exposure level was lower than occupational exposure limits prescribed by China in each type of work, indicating that low-dose omethoate exposure caused the extension of telomere length.

We also analyzed the effects of gender, age, smoking and drinking on telomere length. Considering the influence of age on telomere length, we analyzed the correlation between age and telomere length using the Spearman rank correlation, we found that there was no correlation in the exposure or control group, respectively (*r_s_* = 0.049, *P* = 0.517; *r_s_* = 0.116, *P* = 0.218). Univariate analysis have no found that gender, age, smoking, drinking and working duration had effect on the relative telomere length in the exposure or control group (*P* > 0.05); Multivariate analysis showed that they also had no effect on telomere length (*P* > 0.05), which was consistent with that of univariate analysis.

Genetic injury is the chronic toxic effect of organophosphorus pesticide on the exposure- crowd. The studies in this area are mainly involving in the comet assay and micronucleus test [24–27]. However, genetic injury cannot occur in all individuals who have the same contact condition, suggesting that omethoate-exposure population have different genetic susceptibility. Therefore, this study analyzed the impact of *GSTM1, GSTT1, GSTP1, CYP2E1* and *PON2* genetic polymorphisms on telomere length. The analysis found that the relative telomere length of the *GSTM1* polymorphism deletion genotype was significantly longer than that of the non-deletion genotype in the control group (*P* = 0.004), and the relative telomere length of GG+AG genotypes for *GSTP1* rs1695 polymorphism locus was longer than that of AA genotype in the exposure group. The difference was statistically significant (*P* = 0.024). Liu et al. [28] indicated that individuals with susceptible metabolic *GSTP1* genotypes could experience an increased risk of DNA damage elicited by pesticide exposure, which is similar to the results of this study. Singh et al. [13] also concluded that the GSTM1 null genotype could increase tail moment of the comet assay in workers occupationally exposed to OPs. Gorukmez O et al. [29] found that there was a significant correlation between colorectal cancer and the GSTP1 Ile-Ile genotype when the relationship between GSTT1, GSTM1 and GSTP1 genetic polymorphisms and colorectal cancer was explored. Wong et al [15] researched the relationship between XRCC1, CYP2E1 and ALDH2 genetic polymorphisms and sister chromatid exchange (SCE) frequency and found that CYP2E1 genetic polymorphism was significantly associated with an increased SCE frequency. A large number of studies have shown that variants of rs12026 within the PON2 gene were associated with cardiovascular disease, cerebrovascular disease, diabetes and other diseases [16, 30, 31]. Paraoxonase2 (*PON2*) is a member of the paraoxonase gene family which can protect cells from oxidative stress and thus protect chromosomes from damage. However, the results of this study showed that *CYP2E1*rs6413432, *CYP2E1*rs3813867, *PON2*rs12026 genetic polymorphisms had no effect on telomere length in either group, suggesting that the individual's susceptibility is different to different poisons. Multivariate analysis showed that pesticide exposure (*b* = 0.524, *P* < 0.001) and GSTM1 (*b* = −0.136, *P* = 0.029) were the influencing factors of telomere length, which was consistent with univariate analysis. Therefore, the statistically significant associations between omethoate and telomere length themselves imply the importance of environmental factors such as omethoate and genetic susceptibility in determining telomere length compared with conventional risk factors.

## MATERIALS AND METHODS

### Study population

180 long-term omethoate-exposed workers for longer than 8 years were recruited, and 115 healthy controls were recruited without a history of exposure to OPs or other toxicants, they all lived in the same city. Written informed consent was obtained from each subject, we collected occupational history, basic situation and biological samples for future studies.

### Research methods

### The detection of relative telomere length

Genomic DNA was isolated from peripheral blood lymphocytes, real-time PCR method was applied to determine the relative telomere length, and each sample was run in 3 parallel samples [32].

The PCR was carried out in a volume of 20 μl containing 50 ng of genomic DNA, 2×AceQ qPCR SYBR Green Master Mix 10 μl (Vazyme Biotech co., ltd) and 200 nM each primer (Beijing Invitrogen Corporation). PCR reaction conditions of telomere were as follows: Step1: 15 min at 95°C; Step2: 2 cycles of 15 s at 94°C, 15 s at 49°C; and, Step3: 32 cycles of 15 s at 94°C, 10 s at 62°C, 15 s at 74°C. PCR reaction conditions of reference were as follows: Step1: 15 min at 95°C; Step2: 2 cycles of 15 s at 94°C, 15 s at 49°C; and, Step3: 32 cycles of 15 s at 94°C, 10 s at 62°C, 15 s at 88°C. Data collection was directly completed in the software of ABI 7500 Fast real-time quantitative PCR instrument, and the formula of 2^−ΔΔCt^ (ΔCt=Ct _Telomere_ -Ct_Reference_, ΔΔCt=ΔCt -ΔCt _average normal controls_) was used to calculate the relative telomere length.

### The detection of genetic polymorphisms

Six polymorphic loci of *GSTT1*(*+/*−), *GSTM1* (*+/*−), *GSTP1* rs1695,*CYP2E1* rs6413432, *CYP2E1* rs3813867 and *PON2* rs12026 from five genes were detected using genomic DNA isolated from peripheral blood lymphocytes. Multiplex PCR were used to detect the deletion in *GSTT1* and *GSTM1*; PCR-RFLP method was used to detect other loci for genotyping; primer sequences and restriction endonucleases were described previously [30, 33]. The PCR and enzyme reaction conditions referred to the published paper in task group [34]; just in the experimental process of PCR, we should select a different annealing temperature depending on the Tm value and pre-experimental results.

### Statistical analysis

SPSS21.0 software was used to analyze the data. Methods of representation and examination were based on the distribution of quantitative data. The Spearman rank correlation was used to analyze the correlation between age and telomere length. Using a rank sum test analyzed the effects of genetic polymorphisms on the relative telomere length and the Bonferroni method was used to do the comparisons between the two groups. Multiple linear regression was performed to analyze the influencing factors of telomere length. All statistical tests were two-sided, and the criterion for statistical significance was set at *P* < 0.05 for all tests.

## CONCLUSIONS

In conclusion, the relative telomere length became longer in peripheral blood leukocytes DNA for the omethoate–exposure workers. The length was associated with pesticide exposure and GSTM1 gene polymorphism. The study found that the relative telomere length of the *GSTM1* polymorphism deletion genotype was significantly longer than that of the non - deletion genotype, and the relative telomere length of GG+AG genotype for *GSTP1*rs1695 polymorphism locus was longer than that of the AA genotype for the first time. The research results provide strong evidence for the mechanism research of telomere damage induced by poison and for screening effective susceptible biomarkers.
